# The gauze that time forgot: unmasking a liver mass and the surgical lessons etched in cotton

**DOI:** 10.1093/jscr/rjaf581

**Published:** 2025-07-30

**Authors:** Mario Medinilla, Salvador Lopez-Valdes, Carlos Diaz Q, Douglas Henry

**Affiliations:** Hepato-Biliary and Liver Transplant Surgery Department, Hospital General San Juan de Dios, Guatemala, Guatemala City, Guatemala; Hepato-Biliary and Liver Transplant Surgery Department, Hospital General San Juan de Dios, Guatemala, Guatemala City, Guatemala; Hepato-Biliary and Liver Transplant Surgery Department, Hospital General San Juan de Dios, Guatemala, Guatemala City, Guatemala; Radiology Department, Hospital General San Juan de Dios, Guatemala, Guatemala City, Guatemala

**Keywords:** gossypiboma, retained surgical sponge, intrahepatic mass, surgical complication, hepatic segment resection, foreign-body reaction, open cholecystectomy, surgical safety

## Abstract

Gossypiboma, a retained surgical sponge causing a foreign-body reaction, is a rare but serious postoperative complication. Despite modern safety protocols, such events continue to occur, often underreported due to medicolegal concerns. We report the unusual case of a 23-year-old woman presenting with a palpable upper abdominal mass and systemic symptoms five years after an open cholecystectomy. Imaging suggested a well-encapsulated hepatic lesion in segment 3, raising suspicion for malignancy. Exploratory laparotomy revealed a gossypiboma embedded within liver parenchyma, requiring anatomical resection of segment 3. Histopathology confirmed granulomatous inflammation surrounding retained gauze fibers. The patient recovered uneventfully. This case highlights the importance of considering gossypiboma in the differential diagnosis of unexplained intra-abdominal masses, even years post-surgery. For surgeons, it serves as a critical reminder of the potential consequences of protocol breaches. Reporting such rare complications contributes to awareness, promotes safer surgical practices, and helps prevent recurrence of these avoidable errors.

## Introduction

Textiloma, also known as gossypiboma, is the term used to describe a mass formed by retained surgical materials such as sponges, gauze, or towels. These materials are typically cotton-based and are left inadvertently in the body during surgical procedures. Despite stringent surgical protocols, retained foreign bodies remain a serious and avoidable complication. Incidence rates vary in the literature, with some reports suggesting that textiloma may occur in up to 1 in every 1000 to 1 in every 100 surgical procedures, though this is likely underestimated due to medico-legal underreporting [1].

Clinically, the presentation is highly variable and non-specific, ranging from asymptomatic masses to severe inflammatory reactions, infection, abscess formation, or even fistulas. Diagnosis is often delayed and usually incidental or made through imaging studies or exploratory surgery [2]. The only definitive treatment remains surgical removal of the retained foreign object, which may sometimes necessitate organ resection depending on its location and associated complications [3].

This report presents a case of an intra-abdominal textiloma diagnosed 5 years after an open cholecystectomy, emphasizing the diagnostic challenges, surgical approach, and importance of surgical accountability and vigilance.

## Case presentation

A 23-year-old female, originally from a rural area of Guatemala City, presented with a one-year history of epigastric pain and a palpable abdominal mass. Her medical history was significant for an open cholecystectomy performed five years earlier at a public hospital. The immediate postoperative course had been uneventful. However, over the last year, the patient developed an intermittent, dull abdominal pain localized to the epigastric region, along with occasional nausea, chills, and the subjective sensation of a mass. There was no history of fever, vomiting, jaundice, or bowel habit changes. She did not consume alcohol, smoke, or take oral contraceptives.

Physical examination revealed a hemodynamically stable, thin, and alert patient. Vital signs were within normal limits: blood pressure 117/70 mmHg, heart rate 75 bpm, SpO₂ 98%, and temperature 36.6°C. Abdominal examination showed a mobile mass in the epigastric region, extending to the medial edge of the right hypochondrium. The mass was lateral to the Kocher incision scar and there were no signs of peritoneal irritation. The rest of the physical exam was unremarkable.

Laboratory investigations revealed a hemoglobin of 10.7 g/dl, leukocytosis (14 340/mm^3^) with neutrophilia (86.7%), and elevated liver enzymes (AST 246 U/L, ALT 202 U/L). Tumor markers, including alpha-fetoprotein, CEA, and CA 19–9, were within normal ranges.

**Figure 1:**
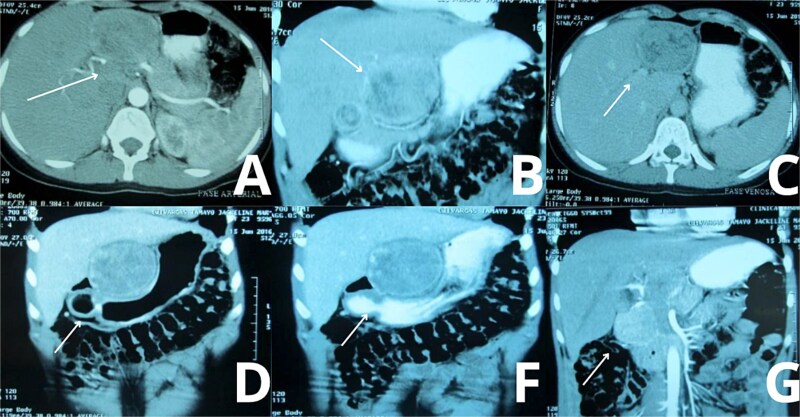
Abdominal CT imaging suggestive of gossypiboma. Contrast-enhanced abdominal CT scans of the patient demonstrate a well-defined, heterogeneous mass located in the upper abdomen, specifically in hepatic segment 3. (A, C) Axial views reveal internal air foci and a linear radiopaque marker, highly suggestive of a retained surgical sponge (gossypiboma). (B) A transverse section further supports the presence of a foreign body, with peripheral enhancement and absence of internal contrast uptake. (D, F, G) Wider axial slices show the spatial relationship of the mass with surrounding hepatic and gastric structures, confirming its intrahepatic location and size (~10 cm), without evidence of ascites or other hepatic lesion.

A contrast-enhanced triphasic abdominal computed tomography (CT) scan demonstrated a 10 cm isodense mass in hepatic segment 3, adjacent to the lesser curvature of the stomach (Fig. 1) The mass exhibited peripheral enhancement but lacked internal contrast uptake. There was no ascites, hepatosplenomegaly, or evidence of other hepatic lesions. The gallbladder was absent, consistent with previous cholecystectomy, and a small fluid collection was seen at the gallbladder bed.

Given the imaging findings and the surgical history, an exploratory laparotomy was scheduled.

### Surgical management

The patient was positioned supine under general anesthesia. A midline supraumbilical incision was made. Central venous access was obtained via the subclavian vein, and a Foley catheter was inserted. Prophylactic antibiotics (Ampicillin-Sulbactam 1.5 g IV) were administered. No arterial line was used.

**Figure 2:**
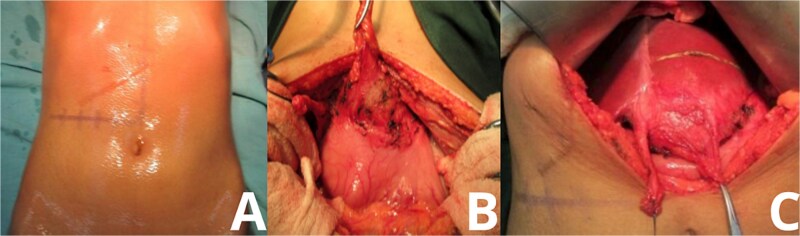
Intraoperative exposure of intrahepatic gossypiboma. Photographs taken during exploratory laparotomy document the identification and mobilization of the gossypiboma. (A) Initial intra-abdominal view reveals dense adhesions between the round ligament and lesser curvature of the stomach. (B, C) Progressive dissection exposes a fibrous, well-encapsulated mass within hepatic segment 3. The mass is separated from surrounding structures with careful technique, avoiding injury to adjacent organs.

Upon entering the peritoneal cavity, dense adhesions were noted between the round ligament and the lesser curvature of the stomach. Careful dissection revealed a well-encapsulated mass located in hepatic segment 3. The mass was tightly adhered to surrounding structures but was eventually mobilized without injury to adjacent organs. The liver parenchyma was marked, and a formal anatomical resection of segment 3 was performed (Fig. 2).

**Figure 3:**
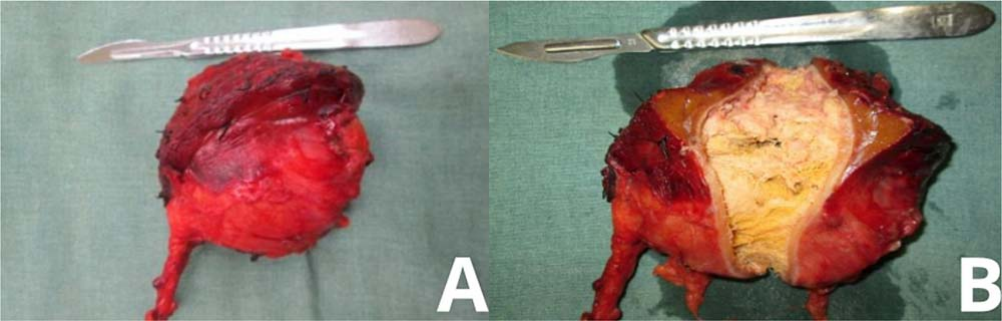
Gross specimen of explanted gossypiboma. Post-resection images of the excised mass show its characteristic features. (A) Lateral view displays the fibrotic outer capsule enveloping the retained surgical sponge. (B) Opposing view highlights the gauze fibers and associated tissue reaction, confirming the diagnosis of textiloma upon histological examination.

The mass measured approximately 10 cm and, upon resection, was found to contain retained surgical gauze consistent with a textiloma. The rest of the intra-abdominal cavity was explored and found to be unremarkable (Fig. 3).

### Histopathology and postoperative course

Histological analysis confirmed the presence of a foreign body granulomatous reaction surrounding cotton fibers, consistent with a retained surgical sponge. No signs of malignancy or abscess formation were observed. The patient’s postoperative course was uneventful. She had no signs of infection, no bile leakage, and was discharged with instructions for regular follow-up. At her one-month postoperative visit, she had resumed daily activities with no further complaints.

## Discussion

Textiloma remains a rare but significant surgical complication. Its presentation depends on multiple factors including the site of retention, size of the material, and the body’s immune response [4]. Some patients may remain asymptomatic for years, while others may develop severe inflammatory reactions leading to abscesses, fistulas, or sepsis. In our case, the retained gauze led to a slowly enlarging hepatic mass that mimicked a neoplastic lesion [5].

The pathophysiology involves a foreign body reaction that leads to the formation of granulomas and fibrous encapsulation around the retained textile material. Two types of responses are described in the literature: exudative (with abscess or fistula formation) and aseptic fibrinous (with granuloma formation). The latter is consistent with our case.

The diagnosis of textiloma is often difficult and usually made incidentally. Imaging modalities like CT or magnetic resonance imaging may reveal suggestive findings, but definitive diagnosis often requires surgical exploration. In our patient, the peripheral capsule enhancement and lack of internal contrast enhancement were consistent with a chronic inflammatory mass rather than a malignancy [6, 7].

From a medicolegal perspective, textilomas raise concerns about surgical quality assurance and documentation. Preventive strategies include rigorous surgical counts, use of radio-opaque sponges, and intraoperative radiography in uncertain cases [8, 9].

This case reinforces the importance of including textiloma in the differential diagnosis of any patient presenting with a mass following prior surgery, even several years postoperatively. Timely diagnosis and complete surgical removal are key to preventing serious complications and restoring patient health.

## Conclusion

This case illustrates the diagnostic and surgical challenges associated with intra-abdominal textilomas. It underscores the necessity of meticulous intraoperative protocols and high clinical suspicion, especially in patients with prior surgical history. Although rare, textiloma should be considered in any patient presenting with an unexplained intra-abdominal mass, particularly in regions where open surgery remains common. Our patient underwent successful resection of hepatic segment 3 containing the retained gauze, with a favorable outcome and return to normal life. Surgical teams must continue to improve safety measures to avoid such preventable errors, thereby safeguarding patient trust and well-being.

## Data Availability

All data supporting this case report are included in the article, with no additional data available due to patient confidentiality.
